# Sample strategies for quantification of hepatic fat fraction mean MRI in healthy cats during body weight gain

**DOI:** 10.1371/journal.pone.0241905

**Published:** 2020-11-12

**Authors:** Francesca Del Chicca, Henning Richter, Gian-Luca Steger, Elena Salesov, Claudia E. Reusch, Patrick R. Kircher

**Affiliations:** 1 Clinic for Diagnostic Imaging, Department of Diagnostics and Clinical Services, Vetsuisse-Faculty Zurich, Zurich, Switzerland; 2 Clinic of Small Animal Internal Medicine, Vetsuisse-Faculty Zurich, Zurich, Switzerland; Universita degli Studi di Padova, ITALY

## Abstract

Hepatic fat fraction (HFF) can be non-invasively estimated with magnetic resonance imaging (MRI) multiple echo gradient recalled echo (GRE) sequence. The aim of this study was to test different methods of sampling strategies to quantify the HFF in healthy cats during body weight gain. Twelve healthy adult male cats were examined in a 3 Tesla MRI unit. Sequences included morphological images, and multiple echo GRE sequence. Cats were scanned at the beginning of the study and twice, each 20 weeks apart during body weight gain. HFF was calculated with 5 different methods of sampling on the multiple echo GRE sequence with different number, size and position of regions of interest (ROIs) and by 2 operators. Results indicated that HFF increased with increasing body weight, and the increase was appreciated with all the 5 methods. There was overall excellent agreement (interclass correlation coefficient = 0.820 (95% confidence interval:0.775–0.856)) between the 2 operators. HFF in the left lateral hepatic lobe was lower than in the other analyzed lobes. HFF measured on large free-hand drawn ROIs was higher than HFF measured with smaller ROIs size. This study proves that different sampling methods for quantification of HFF on multiple echo GRE sequence have overall excellent repeatability and ability to appreciate increased HFF.

## Introduction

Hepatic fat fraction (HFF) in feline population is variable and influenced by the nutritional status, among other factors like diabetes and prolonged fasting [1]. Hepatic fat fraction tends to increase during body weight (BW) gain, overweight and obesity [2]. Increased HFF is present in feline hepatic lipidosis and can cause severe liver disfunction [3].

Increased HFF is difficult to be clinically quantified, and its clinical significance may be variable. Non-invasive techniques to evaluate hepatic fat content in clinical practice are usually limited to ultrasound and computer tomography, but both modalities lack specificity and allow only semiquantitative evaluation of fat content [4]. Assessment of hepatic steatosis for patients care requires not only diagnosis but also grading of severity, and possibility of follow up.

In human medicine, non-invasive quantification of the HFF is routinely performed with dedicated magnetic resonance imaging (MRI) sequences. A substantial number of studies mostly in humans demonstrated that MRI allows a non-invasive, accurate, reproduceable, precise, and reader-independent quantification of HFF regardless the degree of the hepatic lipidosis [4–11].

A recently commercially released multiple echo gradient recalled echo (GRE) sequence (Philips mDIXON-Quant) enables accurate and consistent measurement of the HFF [12]. This sequence allows the assessment of the HFF over the entire liver parenchyma. Correlation of the HFF measured with multiple echo GRE sequence, magnetic resonance spectroscopy and trygliceride quantification has been reported in healthy dogs [13]. No studies have been conducted neither in healthy nor in obese cats. Since cats are prone to pathologies associated with increased HFF, the estimation of HFF, its distribution, and possibility of non-invasive follow up may be beneficial in clinical setting. To the authors knowledge there is no study investigating the sample strategies for a non- invasive estimation of HFF in cats during BW gain mean MRI.

The purposes of the present study are the following: 1) to investigate the difference between 5 sample strategies for the quantification of HFF in cats and to evaluate their diagnostic performance; 2) to investigate hepatic fat distribution during BW gain; 3) to assess agreement between 2 operators with different level of experience, and 4) to investigate the time required for image analysis with the 5 different methods. This will be relevant in clinical non-invasive HFF quantification mean MRI. The present investigation has also the aim to establish a sound method that can be used in clinical patients for the diagnosis of hepatic lipidosis and related hepatopathies, and for the patients recheck following therapy.

## Materials and methods

The prospective, experimental study was approved by the Cantonal Veterinary Office of Zurich (license number, ZH118-16) in accordance with the Animal Welfare Act of Switzerland and as a part of a larger concurrent study. Cats were acquired as kittens, years before, as research animals from a breeding station for research animals (Liberty Research Inc., Waverly New York 14892, USA). Cats underwent MRI examinations at 3 time points: time 0 (T0, at the start of the study before dietary intervention) and twice (T1 and T2, each 20 weeks apart), after the start of dietary intervention.

### Animals

Twelve research purposed-bread, adult, male, neutered shorthair cats were enrolled in this study. All cats underwent a clinical examination. On the basis of a physical examination, haematology and biochemistry, all cats were deemed to be in good health, except two cats with mild elevation of the renal values. All cats had a body condition score of 5/9 at T0. Ten cats were classified as American Society of Anesthesiologists I, the two cats with elevated renal values (International Renal Interest Society, IRIS state 2) were classified as American Society of Anesthesiologists II. One of these 2 cats were excluded from the study before the second MRI examination, and one before the third MRI examination due to causes that are not related to the study.

The BW of the cats was recorded before every MRI examination. The cats received a commercial dry food (Hill’s^TM^ Science Diet^TM^ Adult Optimal Care, Hill’s Pet Nutrition) ad libitum after the MRI examination at T0 for a period of 16 weeks or until they were overweight.

After the 16 weeks, cats received an adjusted amount of feed to keep the BW for the remaining time up to the end of the study. Cats reaching the overweight status (body condition score 7/9) before the 16 weeks period, received an adjusted amount of feed to keep the BW for the remaining time up to the end of the study. The total study length was 40 weeks.

After the completion of the research project, the cats were housed by private families and took no part in any further research.

### Anaesthesia

The cats were fasted for 12 hours before anaesthesia. Premedication consisted of ketamine (10mg/kg), midazolam (0.1 mg/kg) and butorphanol (0.3 mg/kg) intramuscularly. After premedication a catheter was aseptically placed in the left or right cephalic vein for administration of contrast medium, intravenous medication as well as Lactated Ringer’s solution (3 ml/kg/h). Oxygen was administered via a facemask for 30 minutes prior to anaesthesia induction. Anaesthesia was induced with alfaxalone (0.5–2 mg/kg) intravenously. After induction the cats were intubated with a cuffed endotracheal tube and mechanically ventilated with positive-pressure in a pressure-controlled mode (5–11 cmH2O). The respiratory rate was adjusted to achieve an end-tidal CO2 of 35–42 mmHg (4.66–5.59 kPa). The anaesthesia was maintained using isoflurane together with a 1:1 ratio of oxygen and air. Anaesthesia was monitored and recorded with a multiparameter monitor that included spirometry, capnography and an MRI-compatible wireless respiratory sensor, as well as vectorcardiography and pulse oximetry. Glycopyrrolate (10 mcg/kg, intravenously) was administered, if the pulse rate fell below 100 bpm for longer than 10 min. If necessary, this procedure was repeated once.

### MRI protocol

All cats were placed in dorsal recumbency in a 3 Tesla scanner (Philips Ingenia 3.0T scanner, Philips AG, Zurich, Switzerland), with a phased-array anterior coil (dStream Torso, coil solution, 32 channels, Philips AG, Zurich, Switzerland). MRI examination included morphological images to exclude liver abnormalities. Performed sequences were: T2-weighted (turbo spin echo; TR/TE, 2000/80 ms; flip angle, 90°; FOV adapted to animal; voxel size, 1.18/1.42/3.00 mm; slice thickness, 3 mm; slice gap, 0 mm) and T1-weighted pre-contrast sequence (mDixon, gradient echo; TR/TE1/TE 2, 3.7/1.21/2.4 ms; flip angle, 10°; FOV, adapted to animal; voxel size, 1.5/1.5/3.00 mm; slice thickness, 3 mm; slice gap, -1.5 mm).

For the fat quantification, a proton density fat fraction (PDFF), multi-echo acquisition, multi-peak mDixon sequence with T2* correction was performed (mDixon-Quant, Philips AG Healthcare, Zurich, Switzerland). The following sequence parameters were used: breath hold, expiration; TR/TE1/delta TE, 7.5/1.23/1.0 ms; flip angle, 3°; FOV, adapted to animal; slice thickness, 4 mm; slice gap, -2 mm; acquired voxel size, 1.5/1.49/4 mm; echoes, 6. Breath hold technique was used for max. 21.3 seconds. Therefore, controlled mechanical ventilation was discontinued to force brief expiratory apnoea and was continued immediately after the sequence.

T1-weighted post contrast sequence was performed after hand injection of contrast medium (Gadodiamid, GE Healthcare AG, Glattbrugg, Switzerland) (0.3 ml/kg, intravenous) followed by a 10 ml saline (0.9% NaCl) solution: (mDixon, gradient echo; TR/TE1/TE2, 3.7/ 1.21/2.4 ms; flip angle, 10°; FOV, adapted to animal; voxel size, 1.5/1.5/3.00 mm; slice thickness, 3 mm; slice gap, -1.5 mm). All images were acquired in the transverse plane.

### MRI data postprocessing and data analysis

Postprocessing of the multiple echo GRE sequence was performed on the workstation of the previously described MRI unit. HFF was measured on the automatically generated fat fraction images. The HFF was evaluated with 5 different methods of sampling.

**Fig 1:**
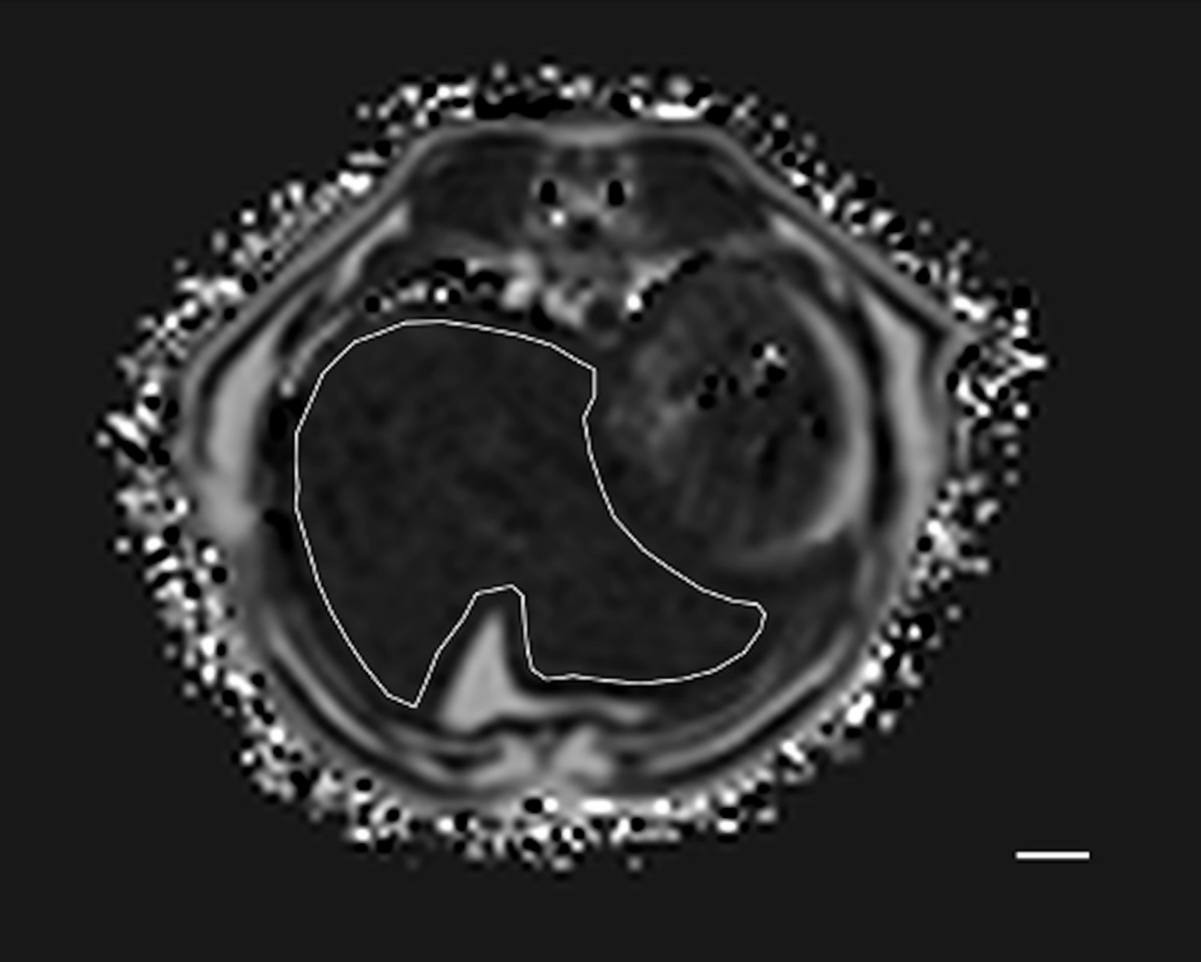
Example of the method 1 of sample strategy (M1). Free-hand ROI was drawn including as much hepatic parenchyma as possible. This was repeated on 10 consecutive slices. In this example cat, at T1, the PDFF was 8.44%. Bar = 1 cm.

Method 1 (M1): ROIs were free-hand manually drawn including as much hepatic parenchyma as possible on 10 consecutive slices. Slices with the most imaged liver parenchyma were selected (Fig 1), with a total of 10 large ROIs. The most cranial and most caudal slices through the liver were avoided, as recommended in human literature [14].

**Fig 2:**
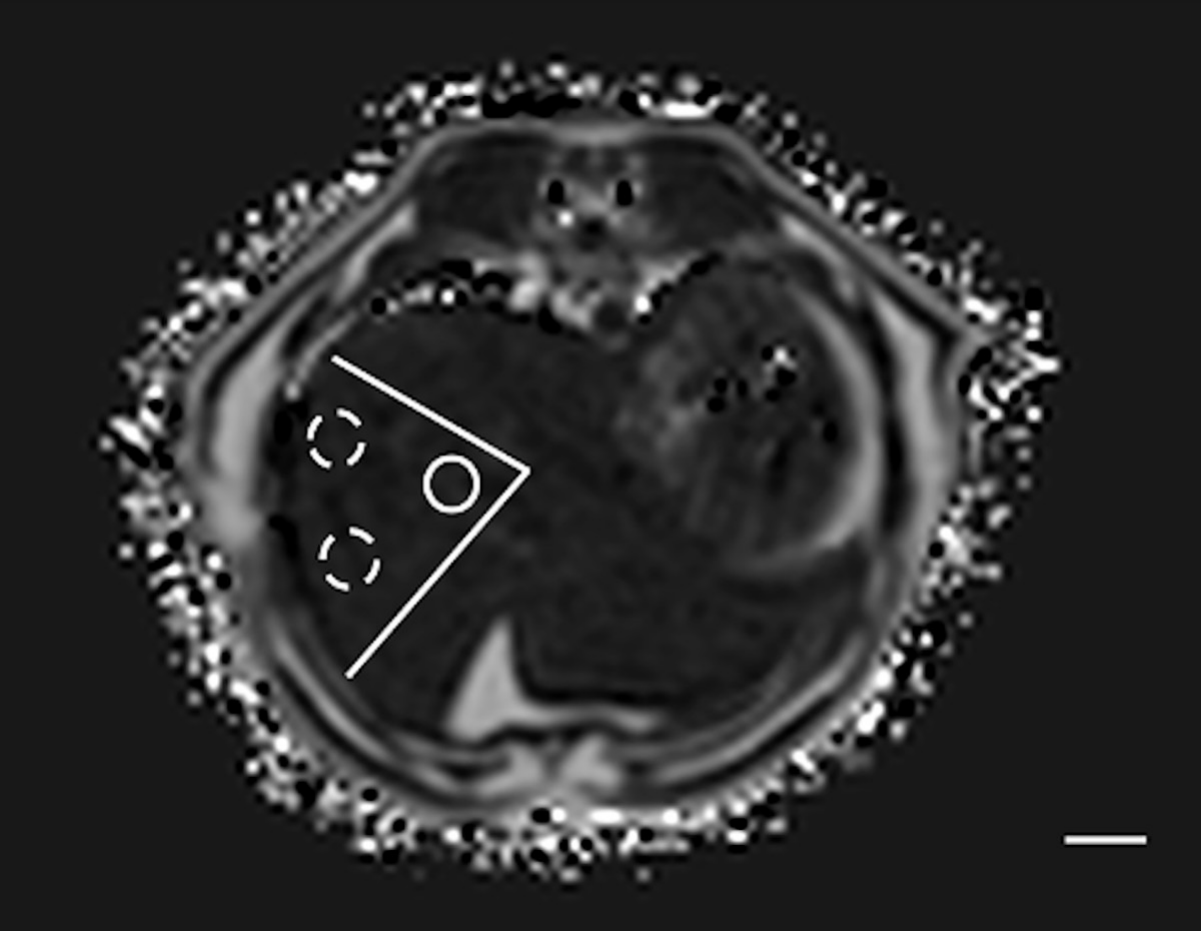
Example of the method 2 of sample strategy (M2). The liver parenchyma was divided in sectors. One round ROI was drawn in the central region (solid line) and 2 in the periphery (dashed lines). This was repeated on 8 slices. In this example cat, at T1, the PDFF in the central region was 9.16%, and in the periphery 7.24 and 7.56%. Bar = 1 cm.

Method 2 (M2): The imaged liver parenchyma, on 8 different slices, was divided in sectors, similarly as described in human medicine [15] and trying to include as much liver parenchyma as possible. One ROI was manually drawn using adjustable round or elliptical cursor in the central part of the sector and 2 ROIs in the periphery, for a total of 24 ROIs per animal (Fig 2).

**Fig 3:**
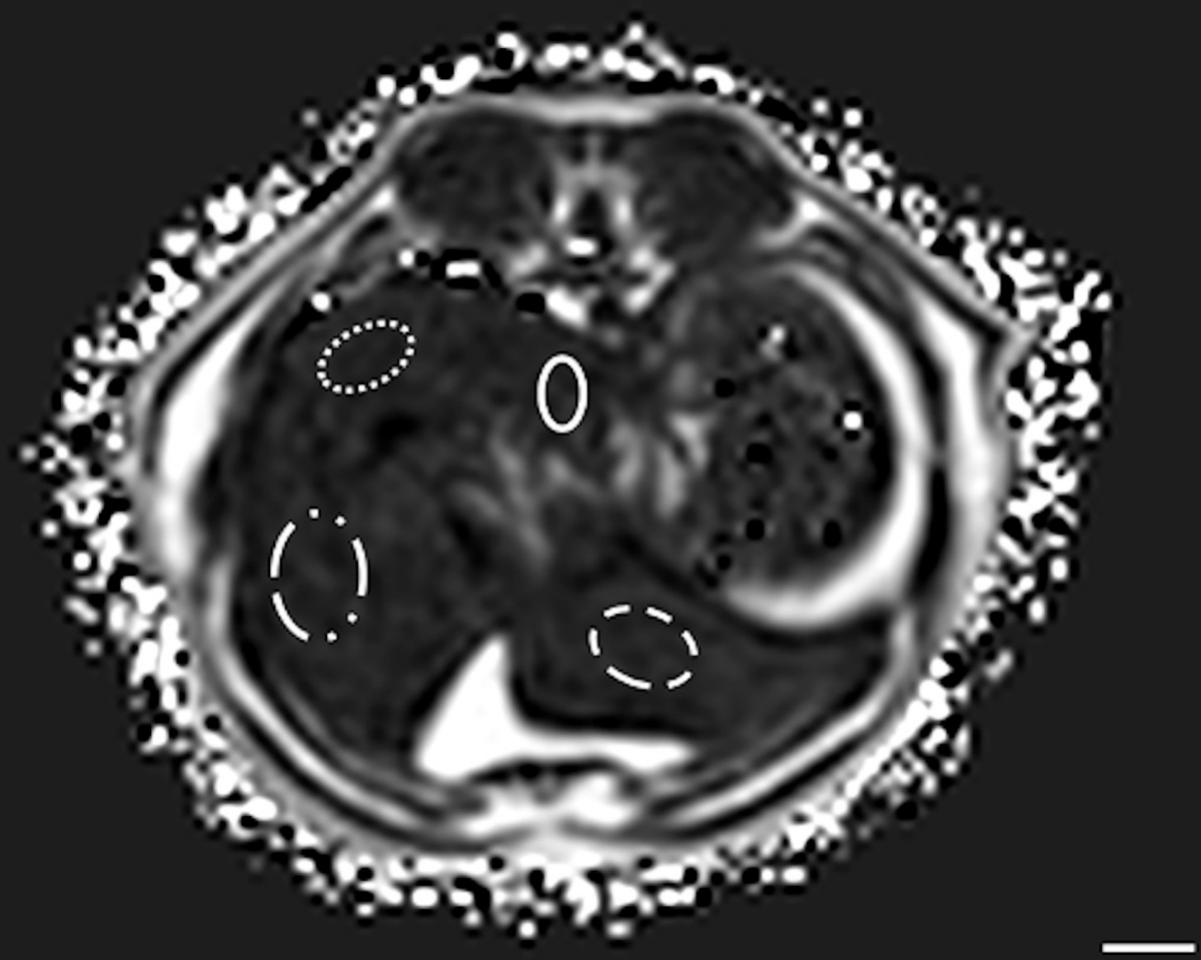
Example of the method 3 of sample strategy (M3). ROIs were drawn on different hepatic lobes. The following hepatic lobes were considered: Caudate lobe (dotted line); right lateral liver lobe (dashed-and-dotted line); papillary process (solid line) and left lateral liver lobe (dashed line). In this example cat, at T1, the PDFF was 2.31% in the caudate liver lobe, 4.38% in the papillary process, 2.06% in the left lateral, and 3.19% in the right lateral. Bar = 1 cm.

Method 3 (M3): One ROI was manually drawn using adjustable round or elliptical cursor in the following liver lobes: caudate (ROI1), papillary process (ROI2), left lateral (ROI3) and right lateral (ROI4) liver lobes as identified [16], on 1 or 2 slices, as necessary for the anatomic identification (Fig 3).

**Fig 4:**
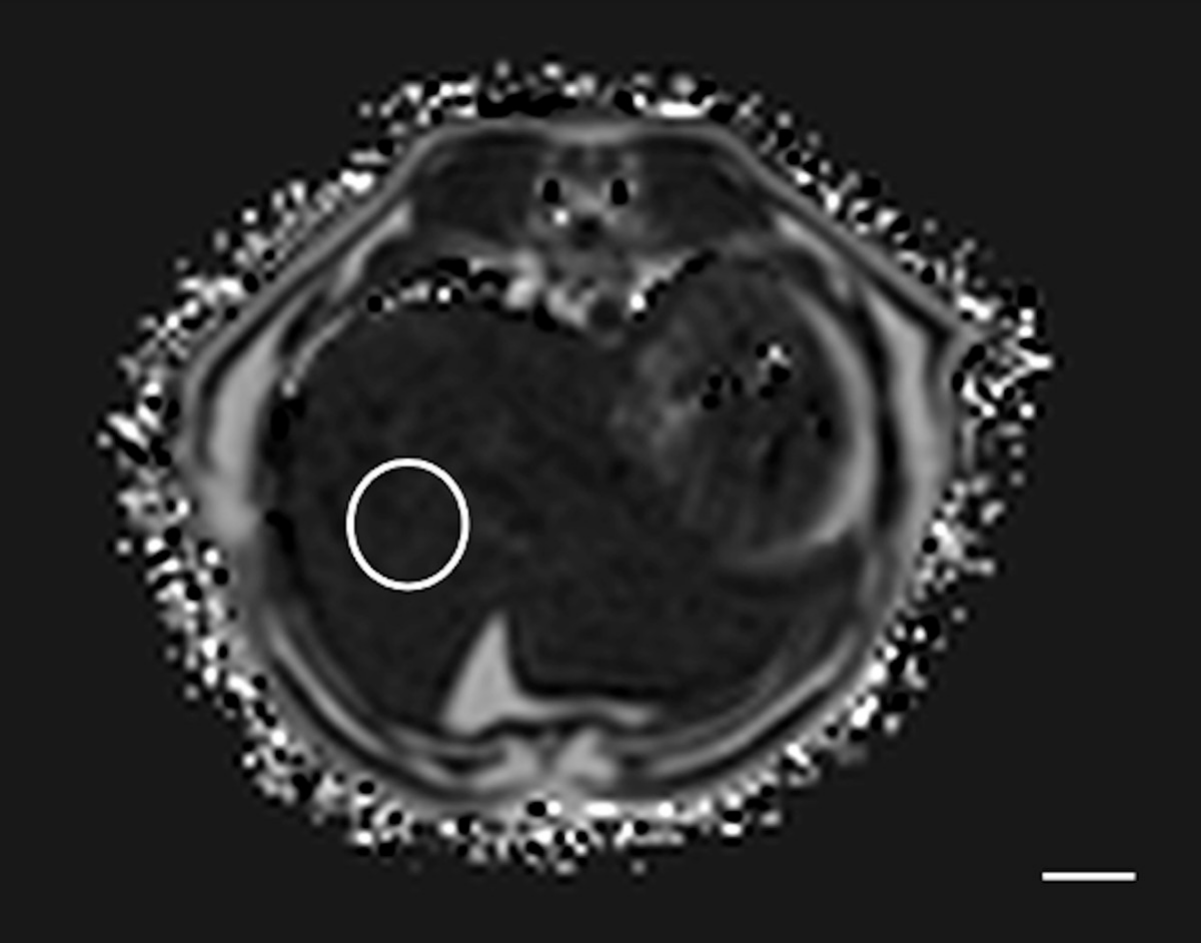
Example of the method 4 of the sample strategy (M4). Large (at least 1cm^2^) ROI was drawn in the right cranial hepatic parenchyma. ROIs of similar size were drawn in the right caudal, middle and left parenchyma. In this example cat, at T1, the PDFF in the right cranial hepatic parenchyma was 4.8%. Bar = 1 cm.

Method 4 (M4): Four ROIs were manually drawn using adjustable round or elliptical cursor in `empiric areas`. One ROI each in the right cranial, right caudal, middle, and left aspects of the liver parenchyma as described [13,17]. ROIs size was at least 1 cm^2^ in diameter and drawn on 4 different slices (Fig 4).

**Fig 5:**
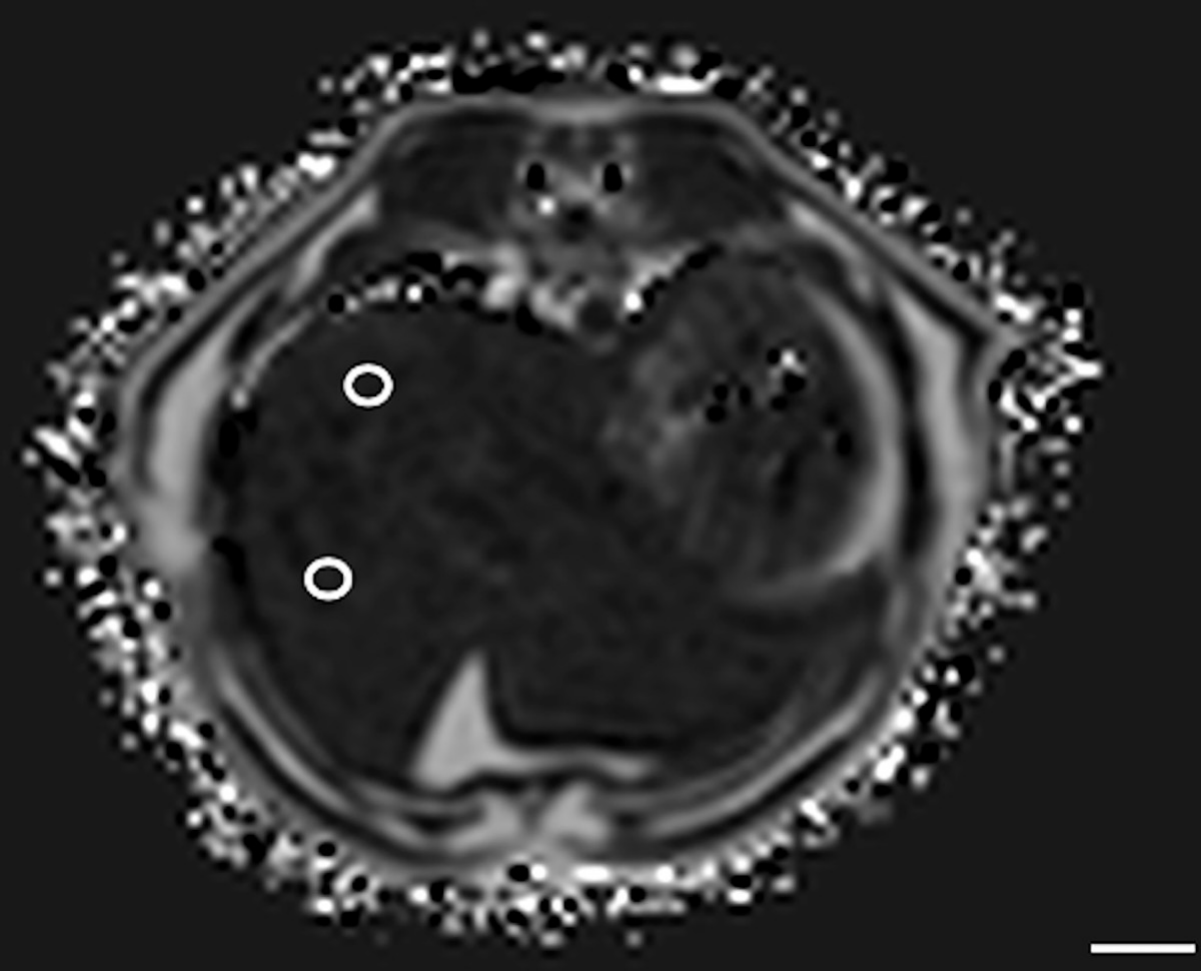
Example of the method 5 of the sample strategy (M5). Small (approximately 0.5 cm^2^ ROIs were drawn throughout the hepatic parenchyma for a total of 16 ROIs. 2 ROIs are imaged. In this example cat, at T1, the PDFF in the imaged 2 ROIs was 7.04% and 10.4%. Bar = 1 cm.

Method 5 (M5): 16 ROIs were manually drawn using adjustable round or elliptical cursor, throughout the liver parenchyma, trying to distribute them randomly throughout the entire organ. ROI size was approximately 0.5 cm^2^ in diameter, and on every slice, a minimum of 1 and maximum of 3 ROIs were drawn (Fig 5).

In all image processing, care was taken to avoid major blood vessels, the gallbladder, and obvious image artifacts during ROIs placement. Two operators independently performed the measurements with all the 5 methods, for each cat, and at T0, T1, and T2. Operator 1 (OP1, FDC) was a radiologist (Dipl ECVDI) with 13 years of experience, while operator 2 (OP2, GLS) was a veterinary doctoral student specifically trained. The time required for the image analysis of each cat and using each different method was recorded, rounded in minutes. ROI size was recorded in cm^2^ as well. Total covered sampled area was obtained by adding the covered area of the single ROIs in each examined liver.

### Statistical analysis

Data were recorded on a computerized spread sheet (Microsoft 140 Excel 2011; Microsoft Corporation, WA, USA). Statistical analyses were performed with a commercially available software package (IBM1 SPSS1 Statistics, version 25, 64-bit-version, IBM, Chicago, Ill). Normal distribution of the data was not assumed due to small sample size. Descriptive statistics were calculated, and numerical data were reported as the mean ± standard deviation for normally distributed data, or as median and range for not normally distributed data. Inter-observer reliability was analyzed with intraclass correlation coefficient (ICC), ranging from 0 to 1.

An ICC <0.4 represented poor agreement; between 0.41 and 0.6 fair; between 0.61 and 0.79 good; and > 0.8 excellent reliability.

Considering the data from OP1, statistical differences among the methods were visualized in Bland- Altman Plots as well as tested with non-parametric tests (Kruskal-Wallis test, Wilcoxon rank-sum test). Tested variables included HFF, time for analysis, and the specific ROI size.

Pearson`s correlation coefficient was calculated between ROI size and HFF at the different time points, and between the total covered sampled area and HFF. Values of *p* < 0.05 or *p*< 0.001 where specified, were considered statistically significant.

## Results

Twelve cats were evaluated at T0, 11 at T1, and 10 at T2. The median age at the beginning of the study was 77 months (range, 75–78 month). Mean of the BW was 4.48 ± 0.44 kg at T0, 6.05 ± 1.02 kg at T1, and 6.35 ± 1.09 at T2.

On morphological images, the liver of all cats was normal on all sequences as described [18]. The mean acquisition time for multiple echo GRE sequence was 14.3 ± 1.7 seconds. On the multiple echo GRE sequence images, a total of 12474 measurements were recorded. ICC between the 2 operators showed excellent agreement over all 5 methods at all 3 time points (ICC = 0.820; confidence interval, CI:0.775–0.856). In particular, M4 showed excellent reliability with the highest ICC value (ICC 0.965; CI:0.930–0.983). The lowest reliability between operators was recorded for M3, (ICC = 0.761; CI:0.593–0.849) still representing good agreement. Among the ROIs of M3, the lowest reliability was recorded for ROI3 (ICC 0.489; CI: -0.233–0.793).

Accordingly, further analyses were performed based on measurements of the most experienced OP1, only.

The measured HFF at T0, T1 and T2 with the different methods are reported in Table 1. The HFF increased with increasing BW as reported [17]. The increased HFF was appreciated with all the 5 methods of image analysis. A concurrent increase of the SD of the measurements over time was recorded for each method. The mean time for image assessment was: 8.67±1.43 min for M1; 5.65±0.89 min for M2; 1.71±0.68 for M3; 1.48±0.26 for M4; and 3.54±0.11 for M5. No statistically significant difference in time required for image analysis was present between M3 and M4 (*p* = 0.24), both consisting of 4 ROIs placement. For all the other methods, the difference in time for analysis was statistically significant considering a cut-off *p* = 0.001 (p<0.001 comparing M1, M2, and M5).

**Table 1:** Mean ± SD HFF in % with the 5 different methods on the 3 time points.

	Method 1	Method 2	Method 3	Method 4	Method 5
Time 0	3.85 ± 0.77	3.87 ± 0.04	3.11 ± 0.85	3.00 ± 0.87	3.37 ± 1.03
Time 1	4.86 ± 1.20	4.31 ± 0.12	3.24 ± 1.37	3.21 ± 0.94	3.64 ± 1.25
Time 2	5.56 ± 1.82	5.13 ± 0.32	4.35 ± 1.83	4.71 ± 2.12	4.69 ± 2.19

M1 was used as reference, because most of the liver parenchyma was covered and included by the measurement. Accordingly, HFF measured with M1 was statistically significant higher (considering a cut-off *p* = 0.001), than HFF measured with M3, (*p*<0.001), M4 (*p*<0.001), and M5 (*p* = 0.004). HFF measured with M2 was statistically significant higher than HFF measured with M3 (*p* = 0.00) and M4 (*p* = 0.005).

**Fig 6:**
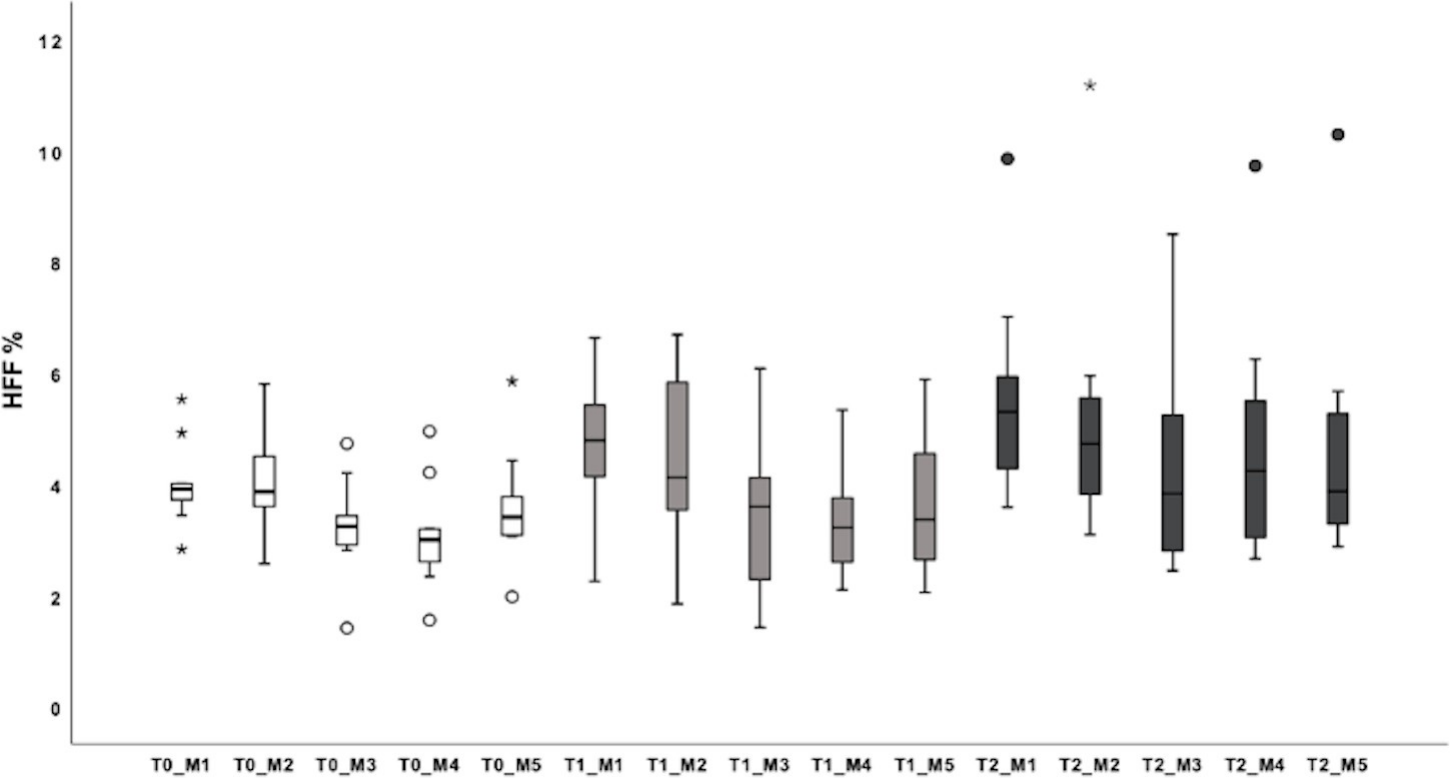
Box plots of the mean HFF measured with the different 5 methods. HFF is reported in % on x-axis. On the y-axis, the different methods of measurements are reported at the different time points: M1, M2, M3, M4, M5 at T0 with white boxes, at T1 with gray boxes, and at T2 with black boxes. For each plot, the box represents the 25^th^ to 75^th^ percentiles, and the dark line represents the median. Whiskers represent the highest case within 1.5-times the interquartile range and the lowest case within 1.5-times the interquartile range. Circles represent the outliers, stars extreme outliers.

ROI3 of M3 showed statistically significant lower HFF than the other ROIs of M3 at all measured time points (*p* = 0.039, *p* = 0.023 and *p* = 0.011 respectively at T0, T1 and T2). ROI2 of M3 showed statistically significant higher HFF compared to ROI3 of M3 on one occasion (at T1, *p* = 0.04). No difference was detected in HFF between central and peripheral areas on M2. HFF measured with the different 5 methods at T0, T1 and T2 is illustrated in Fig 6.

Mean ROI size for the different methods were, respectively: 16.78 ± 1.82 cm^2^ for M1; 0.45 ± 0.02 cm^2^ for M2, 0.48 ± 0.03 cm^2^ for M3, 2.57 ± 0.37 cm^2^ for M4, and 0.43 ± 0.03 cm^2^ for M5.

The total covered sampled area for the different methods was approximately: 167.89 cm^2^ for M1, 10.90 cm^2^ for M2, 1.92 cm^2^ for M3, 10.29 cm^2^ for M4, and 6.94 cm^2^ for M5. No statistically significant correlation was found between ROI size and HFF. Over the 3 time points, total covered sampled area was inversely correlated with HFF for M2 (*p* = 0.037) and M5 (*p* = 0.041).

## Discussion

Non-invasive measurement of HFF and diagnosis of increased HFF is possible with dedicated MRI sequences. We describe 5 different methods of sampling strategies for non-invasive measurement of HFF. All 5 methods were able to detect increased HFF in cats during BW gain in our study population.

Biopsy is currently accepted as the gold standard for determining high fat content in the liver and hepatic lipidosis [9]. Liver biopsy has important limitations: it is an invasive technique that can cause pain, transient hypotension, and other complications such as bleeding, infections, bile leakage, pneumothorax, and hemothorax [19,20], and is not a suitable technique for follow-up evaluations. Moreover, fat accumulations can be heterogeneously distributed across the liver, so that a biopsy sample may not be representative of the pathological processes, and the estimation of hepatic fat content obtained mean biopsy could be inaccurate [20,21]. The severity of hepatic lipidosis is histologically assessed by estimating the percentage of hepatocytes that contain fat droplets. Thus, interpretation of a hepatic biopsy sample is subjective and semiquantitative [22]. As a result, alternative methods to screen for and monitor increased HFF and hepatic lipidosis and inform clinical decision-making are needed [9,20,21,23]. Thanks to chemical-shift-based water and fat separation, Dixon based MRI techniques have been widely used in estimation of HFF and in recent years have been substantially technically improved [24].

While literature agrees on the correlation of the HFF estimated with PDFF with values obtained from other invasive and non-invasive techniques [25], there is no agreement on the methodology of images analysis, with different proposed sample strategies. Results from analysis with ROIs drawn in different locations, shape and number have been evaluated. A common approach is the placement of 9 ROIs of 1 cm in diameter in each of the Couinaud segment of the human liver [26]. This technique is time consuming and requires specific anatomic knowledge, so alternative methods have been investigated. No corresponding anatomical landmarks are described in the feline liver and the hepatic parenchyma is substantially smaller than the human liver. Techniques of investigation have to be specifically tested and adapted for veterinary use.

In humans, the accumulation of fat within the liver tends to be diffuse but the distribution is non-uniform [27]. In particular, PDFF is higher in the right lobe of the liver than in the left [20,28,29], finding confirmed also with liver biopsy [30] and in CT studies [31]. Some other studies reported no difference between the hepatic lobes [32,33] or higher HFF in the left lobe [14]. Interestingly in our study, the HFF in the left lateral liver lobe was statistically significant lower than the other considered liver lobes at all analyzed time points (*p* = 0.039, *p* = 0.023 and *p* = 0.011 respectively at T0, T1 and T2). In humans, it has been suggested that the right liver lobe, supplied by branches of the mesenteric vein, which contains dietary fat, would trigger fat deposition more than the left liver lobe, supplied by the splenic vein [29]. This is consistent with the streamline theory, reported in humans [34], dogs and mice [35]. It is likely that a similar phenomenon is present also in cats and it would explain our results. At the same time, the ROI on the left lateral liver lobe (ROI3 of M3) had the lowest ICC among the 2 operators, suggesting higher variability than other regions.

Another study reports difference in HFF in the peripheral regions compared to the central regions in some hepatic segments in human liver [15]. This has not been confirmed by our data, and no difference was found in the central ROIs compared to the peripheral ROIs at any time of examination (p>0.05). The HFF in the cited human study was markedly higher than in our cat population, ranging from approximately 17 to 21% HFF, reason why direct comparison to the data from our study animals is difficult. It is currently unknown if in cats with hepatic steatosis and higher HFF the distribution of fat could be more heterogeneous than data from the present study. Considering that M2 was also a quite time consuming method of analysis (5.65 ± 0.89 min for animal analysis), this method was not recommended in our study population.

Overall, the SD progressively increased in each method between T0, T1, and T2. This could suggest that progressively increasing HFF tends be more heterogeneously distributed.

Size of the ROIs has also been investigated in human medicine. ROI size of at least 0.75 cm ^2^ have been recommended irrespective of the location [36], and 3 ROIs of approximately 2.3 cm^2^ each (for a total of 6.9 cm^2^ of covered sampled area) have been compared to biopsy results [37]. Another study reported high repeatability using 3 ROIs of 4 cm^2^, each placed on a single image in each of the right posterior segment, right anterior and left medial, for a total of 12 cm^2^ of covered hepatic surface [38]. Other authors [32] recommended sampling of each liver segments in both lobes and sampling a total hepatic area of at least 5 cm^2^.

Recently, sampling covering as much of the hepatic parenchyma as possible using multiple large ROIs has been recommended [39]. On M1, we aimed to sample most of the liver over the entire parenchyma. Using this approach, even if the region of the porta hepatis was excluded drawing the ROIs, part of the perivascular fat tissue may have been included in the analysis, likely resulting in the highest measured HFF.

In our study, the range of covered area ranges from a maximum of approximately 167 cm^2^ for M1, to a minimum of approximately 1.92 cm^2^ for M3. Considering the consistent difference in size between feline and human livers, M1, M2, M4 and M5 sampled overall more of the hepatic parenchyma than often reported in human medicine. The smaller coverage for M3 is clearly explained by the limited ROIs size placed in the caudate lobe and papillary process. The overall coverage of M2 and M4 was similar, but the HFF measured with M2 was statistically significant higher than the HFF measured with M4. A possible explanation could be that more fat tissue was included in the ROIs manually drawn in the centre of the hepatic parenchyma, likely close to the main vessels.

Over time, total covered sample area with M2 and M5 was associated with lower HFF. That would suggest that the use of multiple ROIs (24 ROIs in M2 and 16 ROIs in M5) of similar, small size (in both methods less than 0.5 cm^2^) may be less sensitive in HFF detection and could underestimate the HFF.

Four ROIs sampling strategy (in the anterior, posterior, medial, and lateral segments) has been recommended in human medicine as a reasonable compromise between reproducibility and repeatability, and time invested in the image analysis [39]. This technique also achieved close agreement with the 9 ROIs technique [40]. Four ROIs strategy is also highly reproducible, with ICC >0.9 [38,40]. Our study is in perfect agreement with the published data in human literature, with an ICC = 0.965 for M4. Moreover, M4 was always the least time consuming method, accounting less than 2 min per animal evaluation.

The overall excellent ICC (>0.8) for all methods is particularly noteworthy, taking in account the different level of experience of the 2 operators.

The major limitation of the study is the impossibility to have a gold standard. Trygliceride analysis can not be performed in every location for practical and ethical reason. Moreover, PDFF correlates with chemically determined tissue triglyceride concentration [6] but PDFF and triglyceride concentration obtained with chemical assay measurements are different entities [41]. The measurements of PDFF are an estimation of the true triglyceride hepatic content, which remains unknown. Whether then the HFF is overestimated with M1 or underestimated by the other methods can only be speculated.

Another limitation was the small sample size and limited observation period, both of which were chosen out of consideration for animal welfare. Compared to most of the studies of human medicine which investigate patients with nonalcoholic fatty liver disease, the HFF of our population was lower and the subjects clinically healthy. Further studies conducted over a longer period of time in cats with higher body condition scores and higher HFF, as well as investigations of feline patients with hepatic lipidosis, are needed to assess possible variations in distribution of HFF and the best sample strategy to evaluate these variations. In calculating the time required for image analysis with the different methods, no effect of the learning curve was evaluated. This data were recorded from the analysis of the experienced operator only and we considered the effect of the learning curve similar for each method, and not affecting the overall time difference among the 5 methods.

Despite equipment requirements, high costs, and need of general anesthesia in animals, MRI multiple echo GRE sequence for non-invasive quantification of HFF in cats is very promising as diagnostic and follow up tool.

In conclusions, we suggest a highly reproducible MRI-based method for non-invasive quantification of HFF in cats. The highest reproducibility and the shorter time for analyses were obtained with a 4 ROIs sampling method (M4). The highest HFF was obtained when most of the hepatic parenchyma was free-hand sampled, a method which required the longest time for image analysis. The use of multiple, small ROIs may be less sensitive in HFF detection. The left lateral hepatic lobe has a consistently lower HFF compared to the caudate lobe, papillary process, and the right lateral lobe over time. All the 5 tested methods appreciate an increase HFF during BW gain, and the consistent use of the same sample strategy method is recommended in patients follow up.

## Supporting information

S1 File (XLSX)

S2 File (XLSX)

S3 File (XLSX)
